# Cutaneous metastasis of cecum cancer with MSI-high and BRAFV600E mutation: a case report

**DOI:** 10.1186/s40792-021-01265-w

**Published:** 2021-08-18

**Authors:** Kosuke Yunoki, Takuya Yano, Masanori Yoshimitsu, Ko Oshita, Tetsushi Kubota, Michihiro Ishida, Daisuke Satoh, Yasuhiro Choda, Kanyu Nakano, Yasuhiro Shirakawa, Hiroyoshi Matsukawa, Hitoshi Idani, Shigehiro Shiozaki, Masazumi Okajima

**Affiliations:** 1 Department of Surgery, Hiroshima City Hiroshima Citizens Hospital, 7-33 Motomachi, Naka-ku, Hiroshima, 730-8518 Japan; 2 Department of Gastroenterological and Transplant Surgery, Applied Life Sciences, Institute of Biomedical & Health Sciences, Hiroshima University, 1-2-3 Kasumi, Minami-ku, Hiroshima, 734-8551 Japan

**Keywords:** Colon cancer, Cutaneous metastasis, Microsatellite instability, BRAFV600E, Case report

## Abstract

**Background:**

Cutaneous metastases of colorectal cancer (CRC) are rare, occurring in 0.7% to 5% of cancer patients. Furthermore, the molecular subtypes of cutaneous metastasis of CRC are unclear. Here, we present a rare case of cutaneous metastasis of high-frequency microsatellite instability (MSI-high)/BRAFV600E-mutant cecum cancer.

**Case presentation:**

A 77-year-old woman presented at the outpatient clinic with a subcutaneous mass on her left back. An excisional biopsy was performed and metastatic cutaneous adenocarcinoma was diagnosed. A computed tomography scan of the thorax and abdomen showed thickening of the cecum wall, the presence of pericolic lymph nodes, multiple masses in the liver, and a single nodule in the right lung. Right colectomy with D2 lymphadenectomy and functional end-to-end anastomosis was performed because of the almost-complete intestinal obstruction. The expression of KRAS wild type, BRAFV600E mutation, and MSI-high was detected in the cecum cancer using molecular pathological examination. She received chemotherapy with XELOX + BEV regimen (capecitabine + oxaliplatin + bevacizumab). After four administrations, a computed tomography scan showed reduction of distant metastases, which suggested partial response.

**Conclusions:**

We encountered a rare case of cutaneous metastasis of MSI-high and BRAFV600E-mutant cecum cancer. In the future, it will be necessary to accumulate more cases to identify clinical features and more effective treatments for CRCs with cutaneous metastasis.

## Background

Colorectal cancer (CRC) is a common malignancy worldwide and is one of the leading causes of cancer-related deaths. More than 10% of patients with CRC have metastases at the time of diagnosis. CRC commonly metastasizes to the regional lymph nodes, lungs, liver, and peritoneum, but cutaneous metastases are rare. At the molecular level, CRC is a heterogeneous disease with several molecular subtypes that harbor distinct molecular, genetic, pathologic, and clinical characteristics. Here, we present a rare case of a 77-year-old female with cutaneous metastasis in high-frequency microsatellite instability (MSI-high)/B-type Raf kinase (BRAF) V600E mutant cecum cancer.

## Case presentation

**Fig. 1:**
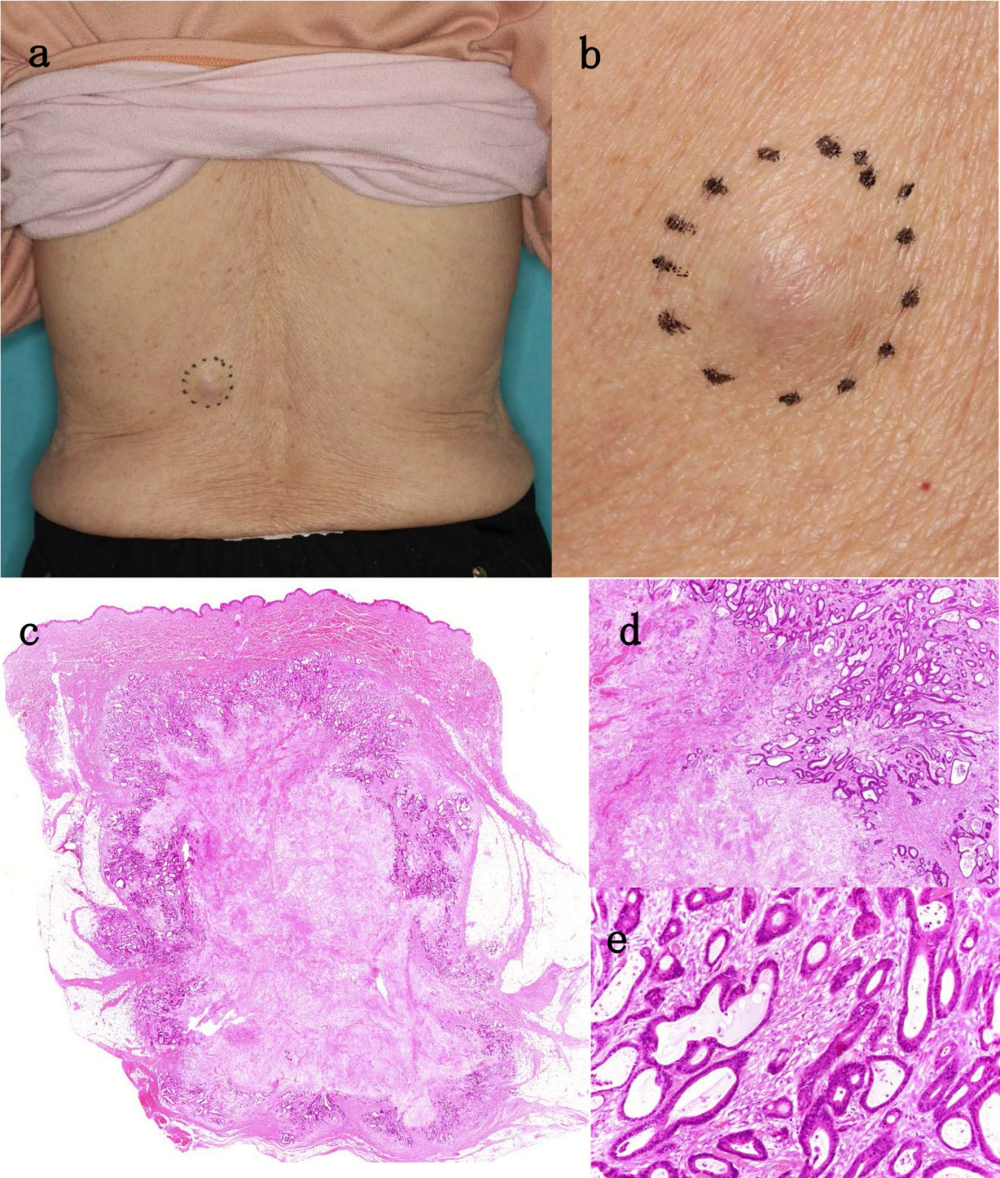
**a**, **b** There was a polypoid lesion with redness on the left back. **c** Lesions were present from the dermis to the subcutaneous fatty tissue. **d** Necrosis was found in a wide area of the lesion. **e** Columnar epithelial cells formed a fused ductal structure. There were also ducts that produce mucus in the lumen (hematoxylin–eosin staining, **c** × loupe. **d** ×20 magnification. **e** ×200 magnification)

A 77-year-old woman presented at the outpatient clinic with a subcutaneous mass on her left back (Fig. 1a, b). She experienced severe weight loss and loss of appetite a few months before visiting the outpatient clinic. Excisional biopsy was performed, and histopathology showed a malignant neoplasm composed of well-formed ductal structures that were growing irregularly and invasively (Fig. 1c–e). Metastatic cutaneous cancer was suspected, because columnar epithelial cells formed a fused ductal structure, mucus was found in the lumen, and extensive necrosis of the lesion was noted. She was referred to our department for further examination and treatment.

**Fig. 2:**
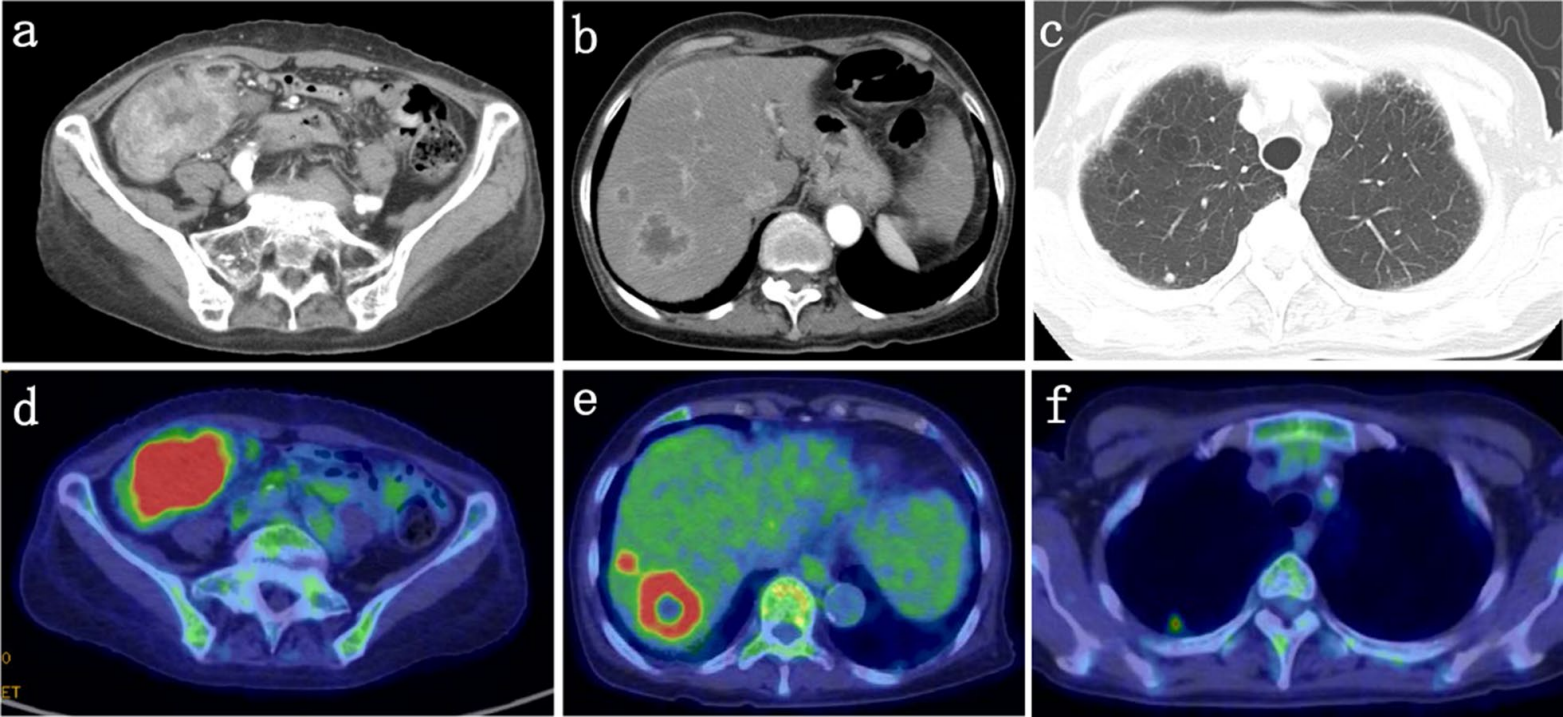
CT scan showed thickening of the cecum wall (**a**) and multiple masses in the liver (**b**), a single nodule in right lung (**c**). FDG-PET/CT revealed elevated uptake in the cecum and masses in the liver and a right lung with a standardized uptake value of 23.7 (**d**) and 8.6/5.9 (**e**), 4.9 (**f**), respectively. CT = computed tomography. FDG-PET/CT = 18F-fluorodeoxyglucose-positron emission tomography/computed tomography

The patient had no significant medical history, allergies, and was not on medication. Laboratory tests showed: Hb 13.4 g/dL, CEA 2.4 ng/mL (normal value < 4.5 ng/mL), CA19-9 < 0.6 U/mL, CA125 128.4 U/mL (normal value < 35 U/mL). Computed tomography (CT) scan of the thorax to the abdomen showed thickening of the cecum wall, the presence of pericolic lymph nodes, multiple masses in the liver, and a single nodule in the right lung (Fig. 2a–c). 18F-fluorodeoxyglucose-positron emission tomography/computed tomography (FDG-PET/CT) revealed increased uptake in the cecum, the masses in the liver, and the right lung with standardized uptake values of 23.7, 8.6/5.9, and 4.9, respectively (Fig. 2d–f). Colonoscopy revealed a circumferential type 2 tumor of the cecum, and endoscopic biopsy showed moderately differentiated adenocarcinoma.

**Fig. 3:**
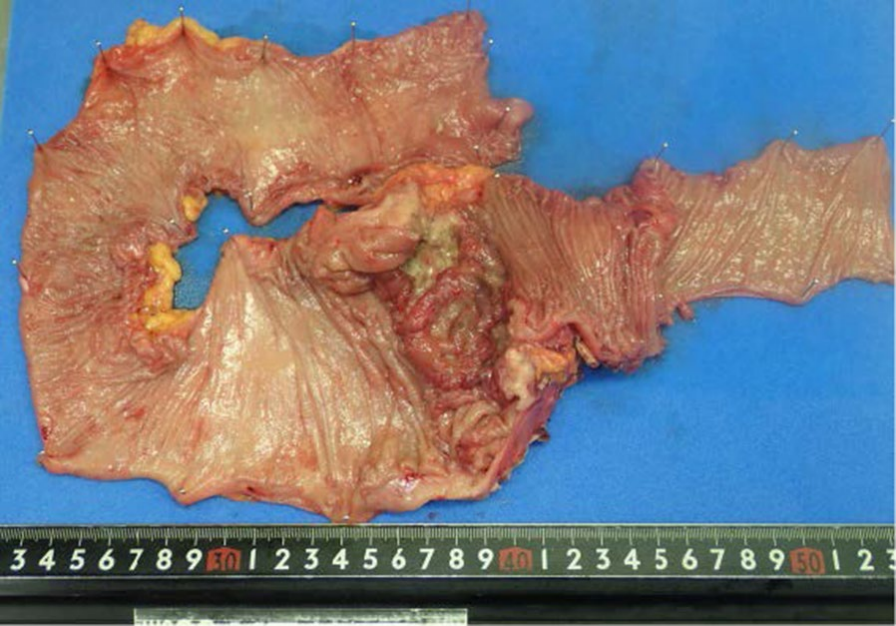
Macroscopically, the tumor was a circumferential type 2 tumor of the cecum

**Fig. 4:**
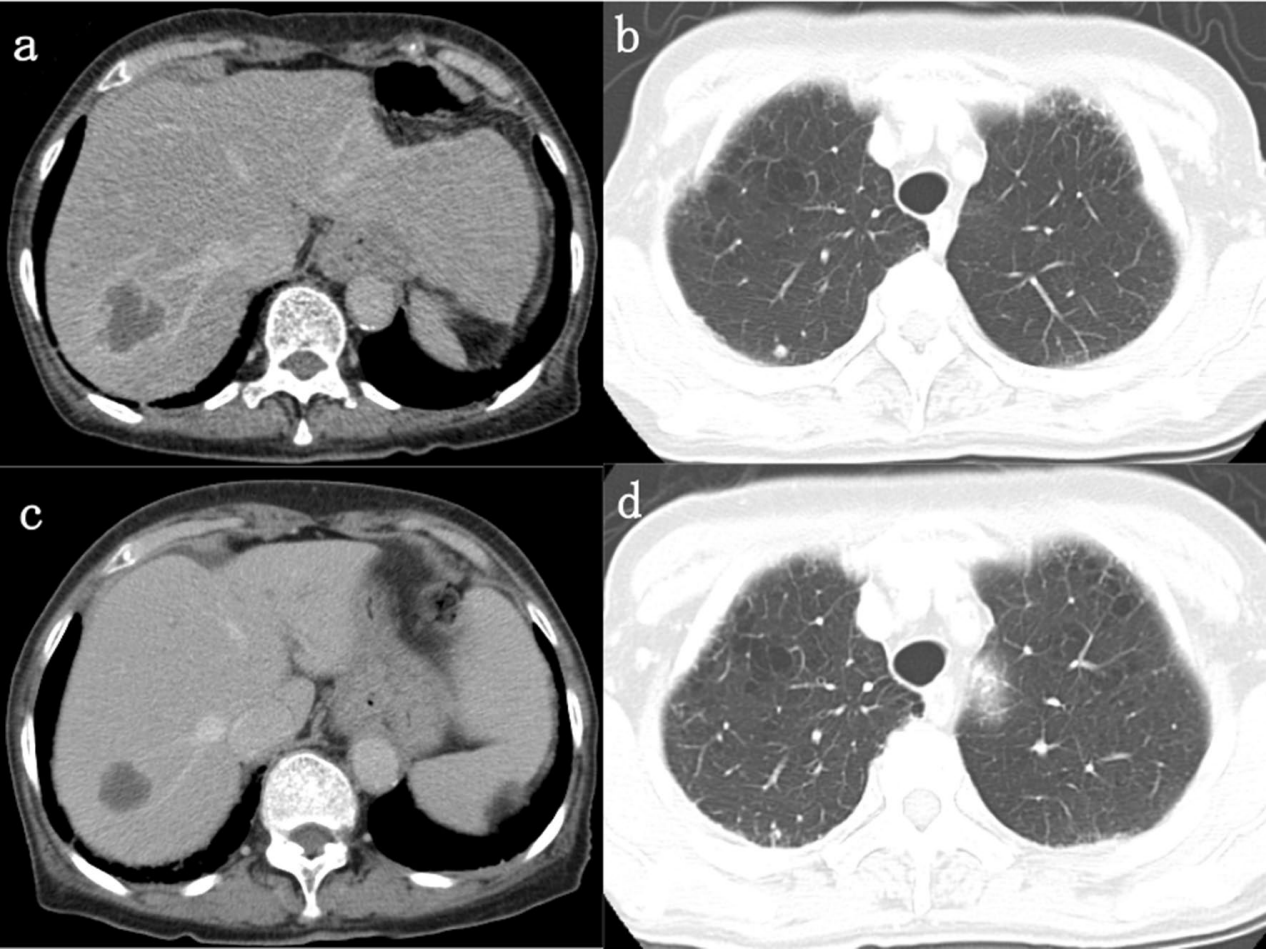
Metastatic mass in the liver before and after XELOX and bevacizumab treatment (**a** and **c**). Metastatic nodule in the right lung before and after XELOX and bevacizumab treatment (**b** and **d**). XELOX = capecitabine + oxaliplatin

Although she presented with unresectable distant metastases, because of the almost-complete intestinal obstruction, right colectomy with D2 lymphadenectomy and functional end-to-end anastomosis was performed (Fig. 3). The pathological diagnosis was moderately differentiated tubular adenocarcinoma with metastases in 5 of 13 resected lymph nodes. Both the primary site and the cutaneous metastatic lesion were not found in these histopathological findings, such as tumor-infiltrating lymphocytes, a Crohn’s-like lymphocytic reaction, mucinous or signet ring differentiation. The final pathological stage was pT4bN2aM1b, Stage IVb according to the Japanese Classification of Colorectal, Appendiceal, and Anal Carcinoma 9th edition. Furthermore, the expression of KRAS wild type, BRAFV600E mutation, and MSI-high was detected in the cecum cancer using molecular pathological examination with the PCR-reverse sequence-specific oligonucleotide probe method. After surgery, the patient received chemotherapy with XELOX + BEV regimen (capecitabine + oxaliplatin + bevacizumab). After four administrations of the XELOX + BEV regimen, a CT scan showed reduction of distant metastases, which suggested partial response (Fig. 4). The patient is alive 8 months after surgery.

## Conclusions

Cutaneous metastasis of cancer is rare, occurring in 0.7%–5% of cancer patients [1]. Higher rates of cutaneous metastasis occur in melanomas, breast and lung cancers, and mucosal carcinomas of the head and neck [2]. The incidence of cutaneous metastasis of CRC is only 2.3% [3]. Furthermore, there are few reports on the genetic background of cutaneous metastasis of CRC. The most common site of cutaneous metastasis in CRC is the abdominal skin, often on surgical incision scars. Other cutaneous sites include the pelvis, back, chest, upper extremities, head, and neck [4]. The exact mechanisms of cutaneous metastasis are still unknown; however, four categories of mechanisms have been reported including the direct extension of primary cancer, lymphatic or hematogenous spread, and surgical implantation [5]. Identification of cutaneous metastasis indicates a poor prognosis. The average survival of patients after the diagnosis of cutaneous metastasis of colon carcinoma is 18 months. In cases of multiple metastases or unresectable lesions, chemotherapy could be considered. When the lesion is resectable and painful, local excision is the preferred treatment option.

MSI is a genetic change caused by a deficiency in mismatch repair (MMR) systems. MSI-high is present in approximately 15% of patients with CRC [4]. MSI-high is more common among those with stage II (20.2%) and stage III CRC (10.9%) [6] but is less frequent among those with stage IV CRC (3.5%) [7]. MSI-high CRC is associated with better survival, right-sided primary tumors, and poorly differentiated tumors with mucinous histological feature [8]. Another report showed that MSI-high CRC is diagnosed at a younger age and has fewer metastases to the liver and lungs than microsatellite stable (MSS) CRC [9]. The most frequent site of metastasis in MSI-high CRC is the perineum (43.8%), followed by the liver (22.9%) and distant lymph nodes (18.8%) [7]. To the best of our knowledge, this is the first report of cutaneous metastasis of MSI-high CRC. MSI-high tumors are heavily infiltrated by activated cytotoxic T-cell lymphocytes and Th1 lymphocytic cells [9]. Pages et al. showed that compared with tumors with vascular emboli and lymphatic and perineural invasion, tumors without these early steps of the metastatic processes had increased infiltration of immune cells [10]. These facts suggest that lymphocytic infiltration may make MSI-high CRC less likely to have distant metastases. There is greater immunoreactivity in deficient mismatch repair tumors [11–13] in stage II/III CRC; therefore, patients with MSI-high tumors have a better 5-year overall survival rate than those with MSS or MSI-low tumors [14]. A previous study showed that lymphocyte infiltration into primary tumors is a strong independent predictor of relapse and overall survival, with high lymphocyte infiltration being a positive prognostic factor in CRC [15]. A Buckowitz et al. reported that MSI-high CRCs with Crohn’s-like lymphocytic reaction are associated with fewer distant metastases [16]. In this case, since there is neither high lymphocyte infiltration nor Crohn’s-like lymphocytic reaction, it may have led to distant metastases. In contrast, MSI-high is a poor prognostic factor in stage IV CRC [17, 18]. Compared with MSS or MSI-low CRCs, MSI-high CRC has the lowest rate of liver metastases and the highest rate of peritoneal metastases, which are related to prognosis [19]. Tran et al. reported that BRAF mutant CRCs were observed to have significantly poorer survival compared with BRAF wild CRCs in MSI-high CRCs [18]. BRAF mutations may contribute to the poor prognosis of stage IV MSI-high CRC.

BRAF is an RAS-regulated serine/threonine kinase in the RAS/RAF/MEK/ERK mitogen-activated protein kinase (M) signaling pathway, which governs proliferation, differentiation, migration, and apoptosis. BRAF mutations are found in approximately 5% of patients with metastatic CRC in our country [20] and 34.6% with MSI-high CRC [17]. BRAF mutation is considered a poor prognostic factor. The most frequent site of metastasis in BRAF-mutant CRC is the liver (63%), followed by distant lymph nodes (56%) and the perineum (46%) [18]. Lianggong et al. reported the first case of cutaneous metastases of BRAF-mutant CRC [21]. In this case, FOLFIRI (irinotecan, calcium leucovorin, and 5-FU) in combination with cetuximab and the BRAF inhibitor vemurafenib caused cutaneous and liver metastases to shrink.

Pembrolizumab [22] and nivolmab + ipilimumab [23] have been confirmed to be effective treatments for MSI-high mCRC. In the KEYNOTE-177 study, pembrolizumab improved progression-free survival in patients with MSI-high metastatic CRC compared with the current chemotherapy regimen for first-line treatment of MSI-high metastatic CRC [24]. For BRAF-mutant metastatic CRC, FOLFOXIRI + bevacizumab was reported as the first-line chemotherapy in the subgroup analysis of the TRIBE study [25]. Furthermore, the effectiveness of the triple therapy of encorafenib, cetuximab, and binimetinib is reported [26]. Chemotherapy including BRAF inhibitors is expected to treat CRC with distant metastases. However, it is unclear whether chemotherapy for BRAF mutations or chemotherapy for high MSI is prioritized for distant metastases with BRAF-mutant and MSI-high CRC. That is the research task hereafter. In our case, XELOX + bevacizumab combination was selected as the treatment because of her poor performance status and based on the domestic drug approval status. It could be one of the treatment options for BRAF-mutant and MSI-high CRC, because the therapeutic effect was confirmed.

We encountered a rare case of cutaneous metastasis of CRC that had MSI-high and a BRAFV600E mutation. In future, it will be necessary to accumulate more cases to identify clinical features and more effective treatments for cutaneous metastatic CRC.

## Data Availability

The data that support the findings of this study are available from the corresponding author, TY, upon reasonable request.

## Abbreviations

CRC: Colorectal cancer
MSI-high: High-frequency microsatellite instability
CT: Computed tomography
FDG-PET/CT: 18F-fluorodeoxyglucose-positron emission tomography/computed tomography, XELOX + BEV: capecitabine + oxaliplatin + bevacizumab, MMR: mismatch repair
MSS: Microsatellite stable
FOLFIRI: Irinotecan + calcium leucovorin + 5-FU
FOLFOXIRI + BEV: 5-Fluorouracil + oxaliplatin + irinotecan + bevacizumab
